# Catecholamine Involvement in the Bioluminescence Control of Two Species of Anthozoans

**DOI:** 10.3390/life13091798

**Published:** 2023-08-23

**Authors:** Laurent Duchatelet, Constance Coubris, Christopher Pels, Sam T. Dupont, Jérôme Mallefet

**Affiliations:** 1 Marine Biology Laboratory, Earth and Life Institute, Université Catholique de Louvain, 1348 Ottignies-Louvain-la-Neuve, Belgium; constance.coubris@uclouvain.be (C.C.); christophe.pels@uclouvain.be (C.P.); jerome.mallefet@uclouvain.be (J.M.); 2 Department of Biological & Environmental Sciences, University of Gothenburg, 451 78 Fiskebäckskil, Sweden; sam.dupont@bioenv.gu.se; 3 Marine Environment Laboratories, International Atomic Energy Agency, MC-98000 Monaco, Monaco

**Keywords:** luminescence, sea pen, *Pennatula*, *Funiculina*, adrenaline, noradrenaline, octopamine

## Abstract

Bioluminescence, the ability of living organisms to emit visible light, is an important ecological feature for many marine species. To fulfil the ecological role (defence, offence, or communication), bioluminescence needs to be finely controlled. While many benthic anthozoans are luminous, the physiological control of light emission has only been investigated in the sea pansy, *Renilla koellikeri*. Through pharmacological investigations, a nervous catecholaminergic bioluminescence control was demonstrated for the common sea pen, *Pennatula phosphorea*, and the tall sea pen, *Funiculina quadrangularis*. Results highlight the involvement of adrenaline as the main neuroeffector triggering clusters of luminescent flashes. While noradrenaline and octopamine elicit flashes in *P. phosphorea*, these two biogenic amines do not trigger significant light production in *F. quadrangularis*. All these neurotransmitters act on both the endodermal photocytes located at the base and crown of autozooids and specific chambers of water-pumping siphonozooids. Combined with previous data on *R. koellikeri*, our results suggest that a catecholaminergic control mechanisms of bioluminescence may be conserved in Anthozoans.

## 1. Introduction

Bioluminescence, defined as the ability of live organisms to produce visible light via biochemical reactions, is an important ecological trait in marine ecosystems [1,2]. Among the benthic sessile organisms, the anthozoan class harbors the most abundant luminous species taxonomically spread over at least 17 different families from four different orders [3]. Among these orders (i.e., Actiniria, Zoantharia, Alcyonacea, and Pennatulacea), Pennatulacea contains the most studied bioluminescent anthozoan species. While physiological studies on the bioluminescence of these species remain anecdotal, the bioluminescence system was extensively documented in many species [3], sea pansy’s (*Renilla* reniformis, *R. muelleri* and *R. koellikeri*) luminous systems being the more investigated (e.g., [4,5,6,7,8,9,10]).

The colonial Pennatulacea luminescence is produced by endodermal cells (i.e., photocytes) spread in the tissue of autozooid and siphonozooid polyps [11,12,13]. Bioluminescence biochemical reactions typically involve an enzyme (i.e., a luciferase) which catabolized the oxidation of a substrate (i.e., a luciferin) with or without the intervention of co-factors [14]. In some cases, pre-oxidized luciferin and luciferase are coupled in a protein complex called photoprotein, needing a cofactor to be activated and produce luminescence [14]. The luminous system of *R. reniformis* involves coelenterazine (i.e., the most widespread luciferin in the marine environment) and the activity of a coelenterazine-dependent luciferase (i.e., *R*Luc) in the presence of dissolved oxygen without other co-factors [5,7]. The *R*Luc was shown to share sequence homology with bacterial haloalkane dehalogenase implying a potential horizontal gene transfer during evolution [15]. Homologous *R*Lucs are found in other Pennatulacea species but also in phylogenetically distinct species such as an echinoderm (i.e., *Amphiura filiformis*; [16]) and a tunicate (i.e., *Pyrosoma atlanticum*; [17]). Evidence tends to demonstrate a similar requirement of both coelenterazine and an *R*Luc homologous luciferase among benthic anthozoans [3,18]. Studies on the sea pansy and other Pennatulacea species also reveal the presence of luminescence-associated molecules such as a coelenterazine binding protein and a green fluorescent protein [19,20,21,22,23,24]. 

The ecological functions of anthozoan light production may provide misdirection for predators, aposematic signals, or burglar alarms [1], although there is no experimental evidence available clearly unveiling these hypotheses. To efficiently perform its ecological role, bioluminescence needs to be finely adjusted and controlled. Bioluminescent organisms possess a variety of control mechanisms to regulate their light emission. Such mechanisms allow organisms the switching of their luminescence on and off and/or the modulation of the physical characteristic of their light emission in terms of color, intensity, or angular distribution [1]. Physiological controls of light emission in marine metazoan species include (i) the direct neuronal and (ii) the hormonal control of the light organ [25,26]. Direct neural regulation is mainly found in intrinsic luminescent organisms and has been extensively studied in osteichthyes and echinoderms. Among the first studied species, Porichthys has been demonstrated as a catecholamine-controlled light emitter [27,28,29,30,31]. Most of the nervous molecules involved in the control of light emission belong to three groups: (i) simple amino acids (e.g., g-aminobutyric acid (GABA), tryptamine or glutamate); (ii) classical neurotransmitters (e.g., serotonin, (nor)adrenaline, acetylcholine, purines, nitric oxide (NO) or octopamine (p-hydroxyphenylethanolamine)); and (iii) neuropeptides (e.g., SALMFamide neuropeptides S1 and S2) (e.g., [25,28,29,30,32,33,34,35,36,37,38,39,40,41,42,43]). 

Previous work suggests some level of phylogenetic conservation in the control mechanisms of bioluminescence. Insects are assumed to mainly use octopamine as a neurotransmitter to control the light emission [32,33]. Acetylcholine is found to be the principal neurotransmitter in ophiuroids [38,44]. The main neurotransmitter of osteichthyes appears to be adrenaline [40,41,42,45]. Nevertheless, some closely related species do not always share common nervous light emission control mechanisms. Nitric oxide has a specific neuromodulator role in neural-induced luminescence since it modulates the light emission in species such as the krill *Meganyctiphanes norvegica*, the midshipman fish *Porichthys notatus*, the hatchetfish *Argyropelecus hemigymnus*, the pearlfish *Maurolicus muelleri* and probably other fishes such as the Myctophidae species [34,46]. Nervous control can either directly control the photocytes or indirectly control the light emission by regulating (i) structural photophore elements (e.g., optical filters, lenses, chromatophores) and (ii) indirect elements linked to photophores (e.g., muscles) [47,48]. Another hormonal physiological control is singularly found in luminescent elasmobranchs [26]. While the majority of bony fish have a nervous control of the emitted light (e.g., via adrenaline, noradrenaline), hormones rather than these neurotransmitters are used for the control of the light emission in luminous shark [26].

Among anthozoans, *Renilla* luminescence is demonstrated to be under an adrenergic control with either rapid flashes triggered at low concentrations (1 pM) or long-lasting glow and superimposed flashes at higher concentrations (1 to 10 mM) [49]. Calcium has also been demonstrated to be needed for the light emission in two anthozoan species, *Renilla reniformis* and *Veretillum cynomorium* [50,51]. No other studies on the bioluminescence physiological control have been published in Pennatulacea.

The aim of this study is to document the physiological control of bioluminescence in two sea pen species inhabiting similar soft sediment ecotypes. The phylogenetic conservation of bioluminescence control among taxa (e.g., octopamine in insects, acetylcholine in ophiuroid, adrenaline in bony fish) leads to the hypothesis that a common control mechanism is shared among anthozoans. As suggested from *Renilla*, this mechanism could have been co-opted from the nervous catecholaminergic control of contractile elements during the pennatulacean evolution [49].

The common sea pen *Pennatula phosphorea* lives in sandy and muddy sediments in the depth between 10 and 100 m. It is distributed throughout the North Atlantic and Mediterranean Sea, with some records from the Indo-Pacific, eastern Pacific, and Southern Oceans [52,53]. This anthozoan possesses a robust axis which may reach 40 cm in length with an emerged rachis up to 25 cm outside the sediment [53]. Autozooid polyps are fused in large triangular structures extending from the rachis in two alternating and opposing rows. This species exhibits rapid flash propagations of green light (maximum emission wavelength (λmax) = 510 nm) along the colony following mechanical stimulations [54,55,56]. Waves of light can be initiated up and down the colony in either direction, highlighting a non-polarized transmission system [56]. As with *Renilla*, the light emission is demonstrated to be under nervous control [11,56,57,58] but without the determination of the neurotransmitters involved. *Funiculina quadrangularis* is a much taller sea pen, exceeding 200 cm in length with approximately one quarter of its body embedded in the sediment [59]. This species is also distributed in fine muddy sediments along the coast of the north-eastern Atlantic Ocean. Each polyp emits a bright blue-luminescence (λmax = 485 nm) in response to a mechanical stimulus [3,60]. While both species may have preferences for different microhabitats within their respective ranges, they share overlapping distribution areas and generally coexist in the same geographic region (i.e., sympatric species).

Our results show that the light production of *P. phosphorea* and *F. quadrangularis* is mainly under a similar nervous adrenergic control. Two other biogenic amines, noradrenaline and octopamine, also play a neuroeffector role in *P. phosphorea* luminescence, but not in *F. quadrangularis*. The catecholamine precursor dopamine is not involved in the bioluminescence control of either species.

## 2. Materials and Methods

### 2.1. Specimen Collection

A total of 50 common sea pens, *P. phosphorea*, were collected in the Gullmarsfjord, Sweden, using a small one-meter aperture dredge at a 35 m depth in May and July 2022. Similarly, 4 tall sea pens, *F. quadrangularis*, were collected in May 2023. Animals were brought back to the Kristineberg Marine Research Station (University of Gothenburg, Fiskebäckskil, Sweden) and maintained in a dark cold room with fresh running deep-sea water pumped from the adjacent fjord. Pictures of the sea pens and their mechanically stimulated bioluminescence were taken with a Sony α7S II camera. 

### 2.2. Dissection

Specimens were anaesthetized by immersion in a MgCl_2_ solution (183 mmol L^−1^ MgCl_2_, 9.9 mmol L^−1^ CaCl_2_, 27.7 mmol L^−1^ Na_2_SO_4_, 20 mmol L^−1^ Tris; pH 8.2) for 30 min. Then, for each tested *P. phosphorea* specimen, (i) the rachis was divided into three equivalent portions defined as upper, middle, or lower, and weighed; (ii) pinnules were dissected, weighed, polyp numbers were counted, and the location on the colony (upper, middle, lower) was defined depending on attachment position on the rachis. Rachis of *F. quadrangularis* was divided into three equivalent portions defined as upper, middle, and lower. Then, each portion was subdivided into multiple 1.5 cm long portions and weighed. For both sea pen species, the peduncle (i.e., the portion of the colony settled in sediment) was also collected and weighed. Sea pen pinnules/rachis/peduncles were rinsed for 3 h in fresh running deep-sea water before pharmacological assays to remove excess of mucus that may impair pharmacological measures.

### 2.3. Evaluation of Luminescence Capability

Measurements of light emission were performed in a dark room using an FB12 tube luminometer (Tirtertek-Berthold, Pforzheim, Germany) calibrated using a standard 470 nm light source (Beta light, Saunders Technology, Hayes, UK). Light responses were recorded using FB12-Sirius PC Software (version 1) (Tirtertek-Berthold). Light emission was characterized as the total amount of light emitted (Ltot) over time, expressed in megaquanta. For *P. phosphorea* pinnules, considering the positive correlation between the numbers of the polyp and the mass of pinnules (Spearman correlation, R^2^ = 0.78, *p*-value < 0.0001; Figure S1A), all data were only standardized per unit of mass (g). For the other sea pen portions (i.e., *P. phosphorea* rachis and peduncle, and *F. quadrangularis* polyp-bearing rachis), data were also standardized per unit of mass (g).

### 2.4. KCl and Pharmacological Assays

Luminescence inductions were performed on pinnules, rachis or peduncle portions placed in a tube luminometer filled with 500 µL of artificial seawater (ASW; 400 mmol L^−1^ NaCl, 9.6 mmol L^−1^ KCl, 52.3 mmol L^−1^ MgCl_2_, 9.9 mmol L^−1^ CaCl_2_, 27.7 mmol L^−1^ Na_2_SO_4_, 20 mmol L^−1^ Tris; pH 8.2). Then, the light emission was triggered with the addition of 500 μL of an KCl depolarizing solution (400 mmol L^−1^ KCl, 52.3 mmol L^−1^ MgCl_2_, 9.9 mmol L^−1^ CaCl_2_, 27.7 mmol L^−1^ Na_2_SO_4_, 20 mmol L^−1^ Tris; pH 8.2), and Ltot was recorded over 3 min [61].

For pharmacological assays, 6-hydroxydopamine hydrobromide (6-OHDA, 162957, Merck, Rahway, NJ, USA) was used as a terminal nervous system catecholamines releaser to confirm the catecholamine implication on the *P. phosphorea* and *F. quadrangularis* luminescence control. *P. phosphorea* pinnules, rachis and *F. quadrangularis* polyp-bearing rachis were placed in a tube luminometer filled with 500 µL of ASW. Light emission was recorded for 30 min after the addition of 500 μL of the 6-OHDA 10^−6^ mol L^−1^ solution [49]. Effects of adrenaline ((±)-Epinephrine hydrochloride, E4642, Merck), noradrenaline (L-Norepinephrine hydrochloride, 74480, Merck), octopamine ((±)-Octopamine hydrochloride, O0250, Merck), and dopamine (Dopamine hydrochloride, H8502, Merck) applications on pinnules and rachis of *Pennatula* and rachis of *Funiculina* were recorded in the luminometer for 30 min. First, adrenaline, noradrenaline, octopamine, and dopamine were tested at a concentration of 10^−6^ mol L^−1^. Dose-dependent response curves (10^−3^ to 10^−6^ mol L^−1^) were then tested for adrenaline, noradrenaline and octopamine on *Pennatula*. For all experiments, the ASW application was used as mechanical (non-pharmacological) control.

### 2.5. Statistical Analysis

All statistical analyses were performed with R Studio (version 2023.03.1 + 446, 2022, R Studio Inc., Boston, MA, USA). Variance normality and equality were tested by the Shapiro–Wilk test and the Levene’s test, respectively. When these parametric assumptions were met, Student *t*-test and ANOVA coupled with Tukey’s test were used, respectively, to perform single or multiple comparisons between groups.

When log transformation did not provide normality and homoscedasticity, non-parametric Wilcoxon test and the Kruskal–Wallis test coupled with the Wilcoxon rank-sum test were used, respectively, to assess whether significant differences were present between two groups or multiple groups. Each difference was considered to be significant at a minimum *p*-value < 0.05. Values were graphically illustrated with mean and standard error of mean (s.e.m).

## 3. Results

### 3.1. Bioluminescence Characterization

**Figure 1:**
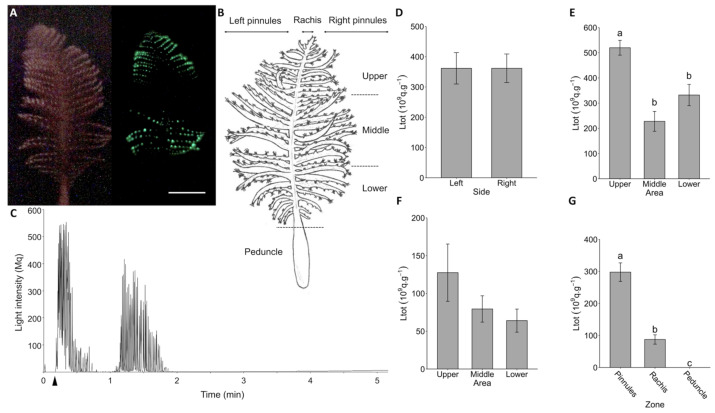
Luminescence capability analyses on *Pennatula phosphorea*. (**A**) Picture of *Pennatula* colony in dim light (left) and emitting waves of luminescence upon a mechanical stimulation (right). (**B**) Representation of a *Pennatula* colony showing the different experimental parts used (left/right—upper/middle/lower, pinnules, the rachis, and the peduncle). (**C**) Original recording of KCl application on pinnules. Arrowhead indicates the KCl application. Total amount of light (Ltot) produced by pinnules under KCl application depending on (**D**) the side of the colony (*n* = 18), (**E**) the position on the rachis (*n* = 12). Ltot produced by rachis under KCl application depending on (**F**) the rachis portion (*n* = 16). (**G**) Comparison between the Ltot produced by pinnules, rachis and peduncle parts of the colony under KCl application (*n* = 50). All Ltot values are expressed as 10^9^ q s^−1^ g^−1^. Different lettering indicates statistical differences. Error bars correspond to s.e.m. Scale bar = 1 cm.

Upon visual observations and video recordings, the luminescence of *P. phosphorea* was confirmed as waves of green luminescence when mechanically stimulated, mainly emitted by autozooid polyps within pinnules (Figure 1A; Video S1). To document the luminescence abilities of the species, colonies were divided into various areas (Figure 1B). Original recordings of the KCl application presented a rapid repetition of flashes for at least 2 min on both pinnules or rachis portions (e.g., Figure 1C and Figure S1B). Light outputs through KCl applications showed no statistical Ltot differences between pinnule sides (Student *t*-test, *p*-value = 0.99; Figure 1D). In addition, the Ltot from the upper part of the colony was significantly higher than either the middle area (Tukey’s test, *p*-value = 0.0001) or the lower area (Tukey’s test, *p*-value = 0.003) (Figure 1E). The Ltot from the middle and lower areas were not significantly different from each other (Tukey’s test, *p*-value = 0.18; Figure 1E). No differences between Ltot were observed between the different rachis portions (Kruskal–Wallis test, *p*-value = 0.65; Figure 1F). The mean Ltot value for pinnules (297.90 ± 28.92 × 10^9^ q s^−1^) was significantly higher than that for the rachis (Wilcoxon sum-rank test, *p*-value = 8.3 × 10^−10^) and the peduncle (Wilcoxon sum-rank test, *p*-value < 2 × 10^−16^) portions (Figure 1G). The mean Ltot value from the rachis portion (87.50 ± 14.55 × 10^9^ q s^−1^) was also significantly higher from the peduncle portion (Wilcoxon sum-rank test, *p*-value < 2 × 10^−16^), which displayed a mean Ltot value of 0.03 ± 0.005 × 10^9^ q s^−1^ (Figure 1G). Moreover, the peduncle part presented values comparable to Ltot values of the ASW control (mean Ltot = 0.41 ± 0.06 × 10^9^ q s^−1^).

**Figure 2:**
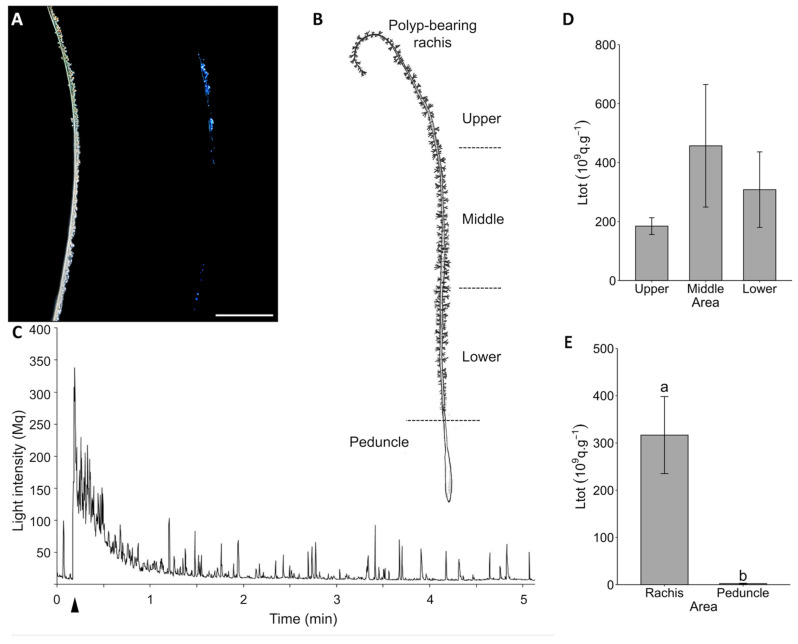
Luminescence capability analyses on *Funiculina quadrangularis*. (**A**) Picture of *Funiculina* colony in dim light (left) and emitting waves of luminescence upon a mechanical stimulation (right) (Dr. E. Infantes pictures). (**B**) Representation of a *Funiculina* colony showing the different experimental parts used (polyp-bearing rachis and peduncle). (**C**) Original recording of KCl application on polyp-bearing rachis. Arrowhead indicates the KCl application. (**D**) Total amount of light (Ltot) produced by polyp-bearing rachis portion under KCl application depending on the position along the colony (*n* = 5). (**E**) Comparison between the Ltot produced by polyp-bearing rachis (*n* = 15) and the peduncle (*n* = 10) parts of the colony under KCl application. All Ltot values are expressed as 10^9^ q s^−1^ g^−1^. Different lettering indicates statistical differences. Error bars correspond to s.e.m. Scale bar = 1 cm.

Similar experiments conducted on *Funiculina* confirmed the production of blue light waves upon mechanical stimulations (Figure 2A). To document the luminescence abilities of *Funiculina*, the colonies were divided into various areas (Figure 2B). The light was emitted by the polyps spread along the rachis. The original recording of the KCl application also showed a rapid repetition of flashes (e.g., Figure 2C). There were no Ltot statistical differences in connection to the location upon the colony (ANOVA, *p*-value = 0.4236; Figure 2D). As shown for *Pennatula*, the mean Ltot value of the polyp-bearing rachis (Ltot = 316.6 ± 81.5 × 10^9^ q s^−1^) was significantly higher than that for the peduncle (Ltot = 2.18 ± 0.81 × 10^9^ q s^−1^; pairwise Wilcoxon test, *p*-value = 3.168 × 10^−5^; Figure 2E). The peduncle part presented values comparable to Ltot values of the ASW control (mean Ltot = 1.72 ± 0.37 × 10^9^ q s^−1^).

### 3.2. Effects of 6-OHDA

**Figure 3:**
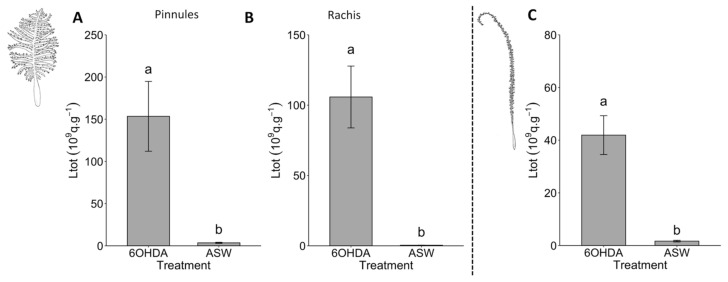
Effects of 6-OHDA 10^−6^ mol L^−1^ on the luminescence of *Pennatula phosphorea* and *Funiculina quadrangularis.* Total amount of light (Ltot) produced after 6-OHDA application on (**A**) the pinnules, and (**B**) the rachis of *P. phosphorea*, and polyp-bearing rachis of *F. quadrangularis* (**C**). All Ltot values are expressed as 10^9^ q s^−1^ g^−1^. Different lettering indicates statistical differences. Error bars correspond to s.e.m. Scale bar = 1 cm.

Application of the 6-OHDA 10^−6^ mol L^−1^ solution on *Pennatula* pinnules resulted in a drastic increase in light production (Ltot = 153.46 ± 41.38 × 10^9^ q s^−1^) compared to the application of ASW (Ltot = 3.51 ± 0.65 × 10^9^ q s^−1^) (pairwise Wilcoxon test, *p*-value < 2.2 × 10^−16^; Figure 3A). Similar results were observed for the rachis with a Ltot value of 105.82 ± 21.98 × 10^9^ q s^−1^ for the 6-OHDA 10^−6^ mol L^−1^ application and of 0.41 ± 0.05 × 10^9^ q s^−1^ for the ASW application (Student *t*-test, *p*-value < 2.2 × 10^−16^; Figure 3B). Similarly, 6-OHDA 10^−6^ mol L^−1^ application on *Funiculina* rachis resulted in an increase in luminescence production (Ltot = 41.9 ± 7.4 × 10^9^ q s^−1^) compared to the ASW control (Ltot = 1.6 ± 0.3 × 10^9^ q s^−1^) (Student *t*-test, *p*-value = 0.0002; Figure 3C). 

### 3.3. Effects of Catecholamines

**Figure 4:**
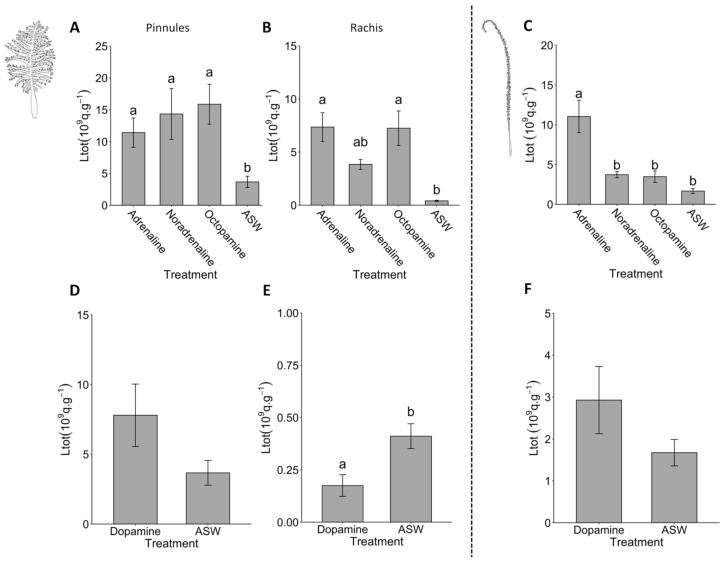
Pharmacological analyses on *Pennatula phosphorea* and *Funiculina quadrangularis* luminescence control. Comparison between the total amount of light (Ltot) produced by (**A**) pinnules (*n* = 62), and (**B**) the rachis (*n* = 20) of *P. phosphorea*, and (**C**) the rachis (*n* = 12) of *F. quadrangularis* after adrenaline, noradrenaline or octopamine applications at 10^−6^ mol L^−1^. Comparison between the Ltot produced by (**D**) pinnules (*n* = 20), and (**E**) the rachis (*n* = 20) of *P. phosphorea*, and (**F**) the rachis (*n* = 12) of *F. quadrangularis* after dopamine application at 10^−6^ mol L^−1^ and ASW control. All Ltot values are expressed as 10^9^ q s^−1^ g^−1^. Different lettering indicates statistical differences. ASW: artificial seawater. Error bars correspond to s.e.m.

Adrenaline, noradrenaline, and octopamine application at 10^−6^ mol L^−1^ generated rapid flash series on the *Pennatula* pinnules that can last for 30 min. Each of those biogenic amines induced the light emission of *P. phosphorea* pinnules compared with the ASW control (pairwise Wilcoxon test, *p*-value < 0.05; Figure 4A), while they did not present statistical differences between each other (pairwise Wilcoxon test, *p*-value > 0.05; Figure 4A). Comparatively, dopamine at 10^−6^ mol L^−1^ did not show statistical differences compared to the ASW application (pairwise Wilcoxon test, *p*-value = 0.13; Figure 4D) on pinnules.

On *Pennatula* rachis, similar results comparing the ASW control to the biogenic amines were obtained for adrenaline and octopamine (Tukey’s test, *p*-value < 0.05), while noradrenaline did not present a significant difference with the ASW control (Tukey’s test, *p*-value = 0.1215; Figure 4B). The response of the three biogenic amine applications did not present any statistical differences between each other (Tukey’s test, *p*-value > 0.05; Figure 4B). Dopamine at 10^−6^ mol L^−1^ did not elicit luminescence of the rachis, with a mean Ltot value significantly lower than the one of ASW (Student *t*-test, *p*-value = 0.0001; Figure 4E). Only adrenaline triggered luminescence flash production in *F. quadrangularis* compared to the ASW control application (Tukey’s test, *p*-value < 2.2 × 10^−6^; Figure 4C), while noradrenaline and octopamine did not differ from the control (Tukey’s test, *p*-value > 0.05; Figure 4C). Dopamine did not trigger light emission in *F. quadrangularis* (Student *t*-test, *p*-value = 0.5603; Figure 4F).

**Figure 5:**
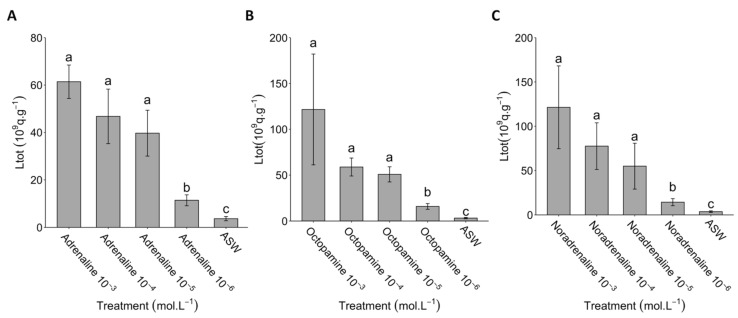
Dose-dependent response of catecholamines on *Pennatula phosphorea* bioluminescence. Total amount of light (Ltot) produced by pinnules after (**A**) adrenaline, (**B**) noradrenaline, and (**C**) octopamine applications at concentrations ranging from 10^−6^ to 10^−3^ mol L^−1^. All Ltot values are expressed as 10^9^ q s^−1^ g^−1^. Different lettering indicates statistical differences. ASW: artificial seawater. Error bars correspond to s.e.m.

At higher concentrations (10^−3^ to 10^−5^ mol L^−1^), adrenaline, noradrenaline, and octopamine application on *Pennatula* pinnules presented an increase in the total light emission compared to the 10^−6^ mol L^−1^ applications (Figure 5; Table S1). At concentrations of 10^−3^ and 10^−4^ mol L^−1^, noradrenaline and octopamine presented a higher response than adrenaline.

## 4. Discussion

The common sea pen, *P. phosphorea* and the tall sea pen, *F. quadrangularis*, produced light after exposure to KCl. The light emission in response to KCl applications confirms not only the bioluminescence status of these species but also the direct involvement of a nervous light emission control [11,56,57,58]. In *Pennatula*’s pinnules, the total light emission did not vary from left to right sides of the colony when the upper part produced a higher luminescence than middle or lower parts. The rachis did not show any statistical differences of total light emission depending on the upper/middle/lower position. Pinnules emitted a more intense luminescence than the rachis which reflected the in vivo observations where only pinnules luminescence has been observed (Video S1). *F. quadrangularis* emitted a homogenous light emission along the axis. For both species, the KCl application on the peduncle part of the colony failed to trigger any luminescence (exceptionally low values like the ASW control). As the function of bioluminescence in these two species is unknown, the biological significance of our observations remains to be clarified. However, as the peduncle is embedded within the sediment, the lack of bioluminescence is not surprising.

6-OHDA is a sympatholytic drug releasing terminal synaptic catecholamines. In both *P. phosphorea* and *F. quadrangularis*, 6-OHDA triggered luminescence, indicating the involvement of catecholamines in the nervous control of light emission. This is consistent with the results of a previous work on *R. koellikeri* [49]. 

In *Renilla*, only adrenaline induces a localized, phasic luminescence at 2 × 10^−4^ M [49]. This is consistent with our observations for *F. quadrangularis.* In *P. phophorea*, the luminescence is shown to be under adrenaline, noradrenaline and octopamine control. An increase in the catecholamine concentration from 10^−6^ to 10^−3^ M results in an increase in the mean Ltot values. All these neurotransmitters induce clustered flashes spaced in time, suggesting a lag time before the next set of flashes. Adrenaline is thought to directly act through the mesogleal nerve net on the β1/β2-like adrenergic receptors of the endodermal photocytes located at the base and crown of the autozooids and specific chambers of the water-pumping siphonozooids of the sea pansy, *Renilla* [35,62]. These catecholaminergic G protein-coupled receptors have been shown to interact with adenylate cyclase to regulate intracellular cAMP concentration [35,62]. The data presented here provide strong support to a similar adrenergic pathway for other luminous pennatulacean species, such as *P. phosphorea* and *F. quadrangularis*. 

Noradrenaline and octopamine neurotransmitters failed to trigger any luminescent flashes in *Renilla* [49] and *Funiculina*. For *P. phosphorea*, noradrenaline at 10^−5^ and 10^−6^ mol L^−1^ induced a similar response to adrenaline in terms of total light emission, while the mean response was higher at 10^−3^ and 10^−4^ mol L^−1^, which suggests a better affinity with receptors (specific or unspecific). Our results also show that octopamine triggers a significative luminous response in *P. phosphorea.* The presence and localization of noradrenaline and its receptor has been detected by high-performance liquid chromatography and immunoreactivity within the mesogleal nerve net of *Renilla* [63]. Conversely, the monoaminergic octopamine molecule has not been detected in the tissues of *R. koellikeri* [63]. In metazoan, noradrenaline, mainly through adrenoceptors, and octopamine, via octopaminergic receptor binding, also act on the cAMP cell concentrations via interaction with membrane-bound adenylate cyclase enzyme [64,65]. The monoaminergic octopamine molecule is structurally close to noradrenaline and known to be involved in the luminescence control in other invertebrates [36], but it is also the main neuroeffector responsible for the light emission in insects [32,66,67,68]. While a study demonstrated the absence of octopamine and its receptors in nerve cells of *R. koellikeri* [63,69], more recent research identified two new aminergic-like G protein-coupled receptors (Ren1 and 2) homologous to mammalian monoaminergic receptors, with cAMP and Ca^2+^-mediated signalling [70,71]. Even if no specific biogenic amines have been identified as specific ligands, adrenaline, noradrenaline and/or octopamine could be assumed as neurotransmitter candidates binding these receptors. Therefore, a cellular pathway involving both noradrenaline and octopamine, their receptors, adenylate cyclase, and cAMP in the light emission control of *P. phosphorea* is here suggested. A similar catecholaminergic control involving both adrenaline and noradrenaline has been demonstrated in bioluminescent bony fishes [40,42,72], highlighting the efficiency of the nervous catecholaminergic pathways to regulate the luminescence and the conservation of such mechanisms among both protostome and deuterostome lineages. However, further investigations are needed to understand the specific affinity of catecholamines with their respective receptors in these luminous anthozoans.

Catecholamines are derived from the tyrosine amino acid [73]. One of the biosynthesis pathways, passing through L-Dopa and dopamine, leads to catecholamines (e.g., noradrenaline and adrenaline; [73] for review). The dopamine failed to trigger light production in both tested species. To date, the dopamine has been described as a weak or non-triggering actor in metazoan luminescence [44,74,75], rather being involved as a light emission inhibitor in some cholinergic-dependent luminous brittle stars [38].

Calcium ions are known to trigger light emission in two anthozoans, *Renilla reniformis* and *Veretillum cynomorium* [50,51], where it is assumed to transiently enter in photocytes breaking down the link between the luciferin-binding protein and coelenterazine that becomes available for the bioluminescent reaction [50,51]. The present results do not provide clues on this aspect, but work is in progress to demonstrate the involvement of calcium ions in *P. phosphorea* and *F. quadrangularis* bioluminescence.

Our results demonstrate biogenic amines to be involved in the light emission regulation in two anthozoan species. Adrenaline acts as the main neuroeffector triggering luminescence in these two species. Further investigations are needed to fully apprehend the pathways and actors involved in the process from the binding of biogenic amines to their receptors till the light production. Based on literature and the presented results, we can assume the conservation of the catecholaminergic control of luminescence among pennatulaceans.

## Data Availability

The original contributions presented in the study are included in the article/Supplementary Materials, further inquiries can be directed to the corresponding authors.

## Supplementary Materials

The following supporting information can be downloaded at: https://www.mdpi.com/article/10.3390/life13091798/s1, Video S1: Original video recording of the light emission of *Pennatula phosphorea* upon mechanical stimulation; Figure S1: (A) Spearman correlation between the number of polyp and the weight of pinnules in *Pennatula phosphorea*; (B) Original recording of KCl application on *P. phosphorea* rachis; Table S1: Statistical analyses of the dose response experiments for (A) adrenaline (Kruskal–Wallis test, *p*-value = 1.69 × 10^−10^; *n* = 6 from 10^−3^ to 10^−5;^ and Wilcoxon test); (B) noradrenaline (Kruskal–Wallis test, *p*-value = 3.96 × 10^−14^; *n* = 6 from 10^−3^ to 10^−5^; and Wilcoxon test), and (C) octopamine (Kruskal–Wallis test, *p*-value = 1.857 × 10^−12^; *n* = 6 from 10^−3^ to 10^−6^; and Wilcoxon test).
