# Supplementary material for: Catecholamine Involvement in the Bioluminescence Control of Two Species of Anthozoans
Source: Life (Basel). 2023 Aug 23;13(9):1798. doi: 10.3390/life13091798 (PMC10533100; doi:10.3390/life13091798)

Figure S1: (A) Spearman correlation between the number of polyp and the weight of pinnules in *Pennatula phosphorea*; (B) Original recording of KCl application on *P. phosphorea* rachis.

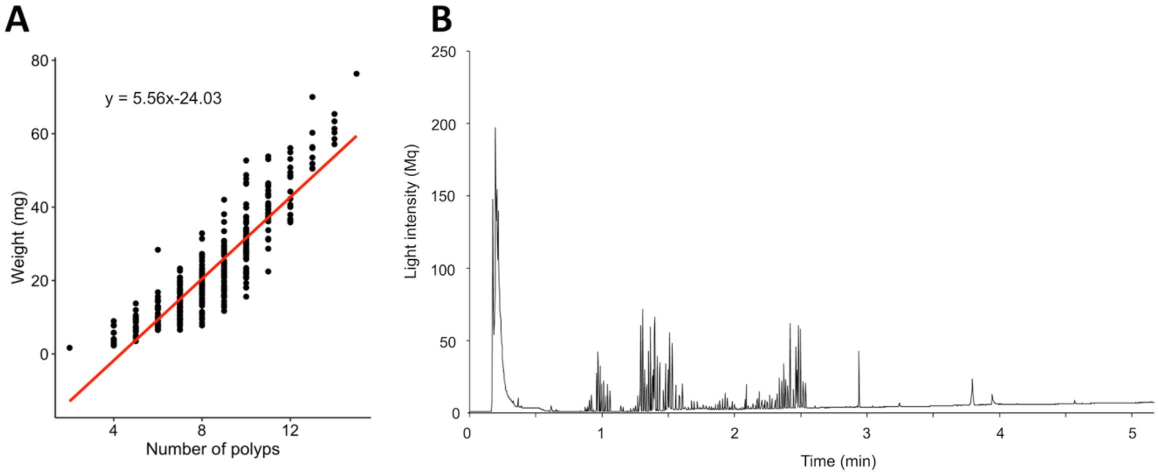

Supplement: Supplementary file 1 [file life-13-01798-s001.zip › life-2566635-Figure S1.pdf]
