# Supplementary material for: Catecholamine Involvement in the Bioluminescence Control of Two Species of Anthozoans
Source: Life (Basel). 2023 Aug 23;13(9):1798. doi: 10.3390/life13091798 (PMC10533100; doi:10.3390/life13091798)
Supplement: Supplementary file 1 [file life-13-01798-s001.zip › life-2566635-Table S1.pdf]

**Table S1:** Statistical analyses of the dose response experiments for (A) adrenaline (Kruskal–Wallis test, df = 4; $\chi^2 = 51.579$, p-value = 1.69 $10^{-10}$; n = 6 from $10^{-3}$ to $10^{-5;}$ and Wilcoxon test); (B) noradrenaline (Kruskal–Wallis test, df = 4; $\chi^2 = 68.853$, p-value = 3.96 $10^{-14}$; n = 6 from $10^{-3}$ to $10^{-5}$; and Wilcoxon test), and (C) octopamine (Kruskal–Wallis test, df = 4; $\chi^2 = 60.92$; p-value = 1.857 $10^{-12}$ ; n = 6 from $10^{-3}$ to $10^{-6}$; and Wilcoxon test).

## A

| Adrenaline | $10^{-3}$ | $10^{-4}$ | $10^{-5}$ | $10^{-6}$ | ASW |
|---|---|---|---|---|---|
| $10^{-3}$ | / | / | / | / | / |
| $10^{-4}$ | 0.6191 | / | / | / | / |
| $10^{-5}$ | 0.3961 | 0.6191 | / | / | / |
| $10^{-6}$ | 0.0016 | 0.0042 | 0.0066 | / | / |
| ASW | 0.0007 | 0.0009 | 0.0009 | 0.0009 | / |

## B

| Noradrenaline | $10^{-3}$ | $10^{-4}$ | $10^{-5}$ | $10^{-6}$ | ASW |
|---|---|---|---|---|---|
| $10^{-3}$ | / | / | / | / | / |
| $10^{-4}$ | 0.9697 | / | / | / | / |
| $10^{-5}$ | 0.7208 | 0.9697 | / | / | / |
| $10^{-6}$ | 0.0043 | 0.0043 | 0.0043 | / | / |
| ASW | 0.0007 | 0.0007 | 0.0007 | $<0.0001$ | / |

## C

| Octopamine | $10^{-3}$ | $10^{-4}$ | $10^{-5}$ | $10^{-6}$ | ASW |
|---|---|---|---|---|---|
| $10^{-3}$ | / | / | / | / | / |
| $10^{-4}$ | 0.7879 | / | / | / | / |
| $10^{-5}$ | 0.5390 | 0.7879 | / | / | / |
| $10^{-6}$ | 0.0015 | 0.0033 | 0.0083 | / | / |
| ASW | 0.0006 | 0.0007 | 0.0009 | $<0.0001$ | / |
